# Intergenerational Food Insecurity, Underlying Factors, and Opportunities for Intervention in Momostenango, Guatemala

**DOI:** 10.3390/nu16040470

**Published:** 2024-02-06

**Authors:** Ginny Lane, Silvia Xinico, Michele Monroy-Valle, Karla Cordón-Arrivillaga, Hassan Vatanparast

**Affiliations:** 1 Margaret Ritchie School of Family and Consumer Sciences, University of Idaho, Moscow, ID 83843, USA; 2 Alianza Nacional de Organizaciones de Mujeres Indigenas por la Salud Reproductiva Nutrición y Educación (ALIANMISAR), Guatemala City 04001, Guatemala; silviaxinico@gmail.com; 3 School of Public Health, University of Saskatchewan, Saskatoon, SK S7N 2Z4, Canada; m.monroyvalle@usask.ca; 4 Unidad de Investigación en Seguridad Alimentaria y Nutricional (UNISAN), Escuela de Nutrición, Facultad de Ciencias Químicas y Farmacia, Universidad de San Carlos de Guatemala, Guatemala City 01051, Guatemala; krcordon@gmail.com; 5 College of Pharmacy and Nutrition, University of Saskatchewan, Saskatoon, SK S7N 5E5, Canada

**Keywords:** food insecurity, climate change, food production, Momostenango, Guatemala

## Abstract

Achieving sustainable food security in Guatemala, where nearly half the population is food insecure and 50% of children face chronic malnutrition, is challenging. This mixed-methods study aimed to identify the impacts of climate change on food production, community food security, and household food security. Twelve agricultural group leaders in six communities were interviewed using semi-structured guides. Key informant interview themes included subsistence agriculture, commercial production, challenges related to climate, capital, market, and capacity, as well as sustainable opportunities. Fifty-five mothers from 13 distinct communities around Momostenango were surveyed and interviewed. A significant finding is that 85% of households were food insecure, with 93% relying on agriculture. Food-secure families mostly worked on their own or leased land, whereas food-insecure ones combined farming with day labor. In times of food scarcity, strategies such as altering food consumption and reducing expenses were common. Severely food-insecure families were significantly more likely to reduce portion sizes (72%), whereas food-secure families typically resorted to less preferred foods. Overall, food insecurity was notably linked to larger families, older mothers with limited education, and reliance on agricultural day labor. Food insecurity is a long-term issue in rural areas, deeply rooted in structural socioeconomic constraints, and recurring across generations.

## 1. Introduction

Progress towards sustainable food security remains a formidable global development challenge. In 2022, almost one quarter (23%) of the Latin America and the Caribbean population could not afford a healthy diet [1]. This challenge is particularly pronounced in Mesoamerica (Costa Rica, Nicaragua, Honduras, El Salvador, Guatemala, Belize, and central to southern Mexico), where 28% could not afford a healthy diet, and in the Caribbean, where this figure rose to 52%. Across Latin America and the Caribbean there is a clear correlation between the inability to afford a nutritious diet and various socioeconomic indicators, including national income level, poverty incidence, and income inequality. The surge in international food prices since 2020, further intensified by the conflict in Ukraine, and coupled with an additional regional increase in food inflation, has exacerbated difficulties with accessing a healthy diet across the region.

Rural poverty and food insecurity are endemic to Guatemala, where more than 45% of households are considered moderately or severely food insecure [2]. Guatemala has the highest level of chronic child malnutrition (47%) in the Western hemisphere and the sixth highest level in the world [3]. A high prevalence of “hidden hunger” also exists in Latin America, where individuals consume sufficient calories, but the diet is deficient in micronutrients [4], such as the 32% of Guatemalan children under the age of five that are anemic [5]. Many Guatemalan households are experiencing the double or triple burden of malnutrition characterized by chronic child malnutrition, anemia, and maternal obesity, where 39% of chronically malnourished Guatemalan children have overweight or obese mothers [5].

Food insecurity is especially precarious for populations living in the Central American Dry Corridor (CADC), where a series of abnormal weather events, including drought and extreme precipitation, have contributed to increased food insecurity amongst the rural population over the last decade [6]. Rural families are particularly vulnerable to seasonal hunger, typically during April–August when stored food or income from previous harvests has been used up, funds are diverted to purchase fertilizer for the new growing season, and peak demand for agricultural labor has passed [7]. The El Niño extreme weather event in 2014–2015 caused an estimated 80% crop loss in Guatemala [8]. In addition, there is historical resistance to colonization by Indigenous leadership in Totonicapán that puts them at higher risk of food insecurity. This strong group of Indigenous leaders seeks to protect the interests of Mayan peoples. Unfortunately, in response to this ongoing resistance, the national government does not prioritize the area for implementation of social services [9].

Recently, high food insecurity in the dry corridor has been accompanied by a surge of migration to the United States from Central America, resulting in a significant spike in the number of apprehensions at the U.S border, along with an increasing proportion of unaccompanied minors and women with children [10]. Migrants from the dry corridor commonly cite poverty and unemployment as reasons for emigration, followed by agricultural losses and adverse climate events.

**Figure 1:**
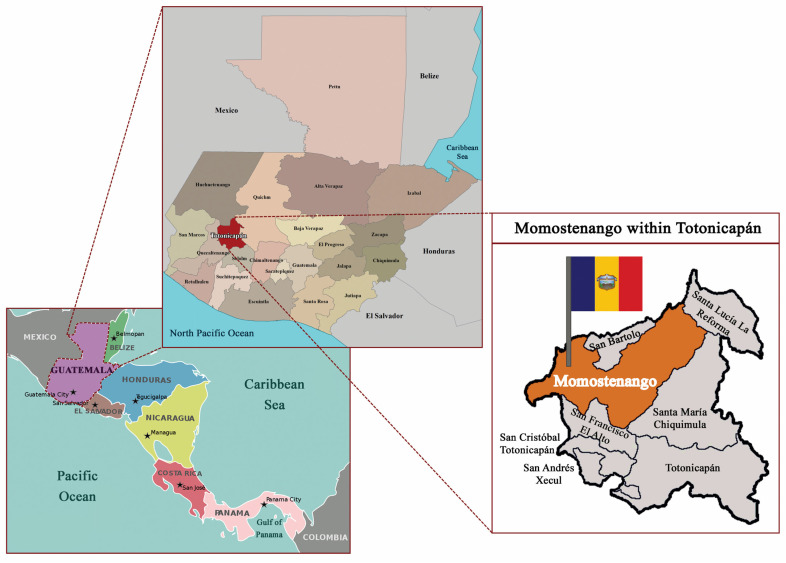
Map of Momostenango, Totonicapán, Guatemala.

The department of Totonicapán (Figure 1), including the municipality of Momostenango, located on the edge of the dry corridor, is home to the highest proportion of Mayan people in Guatemala (98%) and is one of poorest areas of Guatemala [11]. While there are not statistics that state the average income of Mayans compared to the non-Indigenous population in Guatemala, Mayans in rural areas often work as day laborers in the agricultural sector, one of the poorest paid occupations, above only domestic workers [12,13]. In addition, extreme poverty affects 21.8% of the Indigenous population, compared to 7.4% of the non-Indigenous (Mestizo) population [14]. Totonicapán is one of the five departments rated as chronically severely food insecure, meaning that a majority of households do not consume sufficient calories for more than four months per year, resulting in a nutritionally inadequate diet over the whole year [15]. Seventy-five percent of Totonicapán residents are considered moderately or severely food insecure. In 2018, 72% of the population were small farmers with 0.04–0.12 hectares of cultivated land, 15% were day laborers with no land, 12% were commercial farmers with 0.16–0.83 hectares of land, and 1% were classified as commercial export farmers with more than 1 hectare of land. Migration in search of employment commonly occurs, with permanent migration to Guatemala City and the United States and temporary migration to coffee plantations in Guatemala and Chiapas, Mexico. Remittances from family members that have emigrated are an important source of income for many families living in Totonicapán [15]. 

Given the serious gravity of chronically high food insecurity in Totonicapán resulting in malnutrition and migration, there is a need to understand how to successfully intervene to build sustainable community food security. The current study employs a mixed-methods design to comprehensively understand local food insecurity and opportunities to intervene.

## 2. Materials and Methods

The exploratory sequential mixed-methods study design included qualitative key informant interviews to identify the impacts of climate changes on food production and community food security, as well as quantitative family interviews to gather details about socio-demographics and household food security.

### 2.1. Sample and Recruitment

The target study population resided in the municipality of Momostenango, department of Totonicapán, with the majority living in rural areas surrounding the city of Momostenango. In both the qualitative and quantitative components, the snowball method was used to recruit key informant and family participants. An agricultural extensionist and community food security advisory committee arranged meetings with the key informants, including agricultural extensionists, community leaders, and women’s group leaders. These key informants then reached out through their community networks in 13 distinct areas to invite families to participate.

A priori samples size was calculated using G power [16]. In order to observe a large effect (0.5), using an alpha of 0.05 and a power of 0.8, a sample of 52 family interview participants is required to test for goodness of fit of the food-secure group compared to the moderately food-insecure and severely food-insecure groups.

### 2.2. Quantitative Data Collection Tool

Families were interviewed using a modified version of the National Maternal-Infant Health Survey of Guatemala questionnaire (ENSMI) in Spanish [5]. This questionnaire has been validated previously in the field [17]. The questionnaire was shortened to reduce participant burden by removing sections related to reproductive health and domestic violence that were not required for the study. The questionnaire included questions related to demographics, agricultural engagement, food security, coping strategies, food use during times of scarcity, and preferred food security interventions.

### 2.3. Qualitative Data Collection Tool

A semi-structured Spanish interview guide was developed to gather qualitative data about community food security and the impact of climate change from key informants. The questions were derived from a review of the recent literature and consultations with the community food security committee. The key informant interview guide included questions related to crops commonly cultivated, current agricultural challenges, food security, and perspectives on how food security could be improved. 

### 2.4. Qualitative and Quantitative Data Collection

Data collection took place between July and November 2018. The Spanish-speaking lead research team member conducted all key informant interviews, lasting approximately one hour, at community gathering places or their homes. The researcher took detailed notes and translated them into English prior to analysis. Interviews were conducted until further interviews did not yield additional information beyond that already collected, indicating saturation had been reached after 10 interviews [18]. Data saturation can usually be achieved with 8–12 interviews [19]. The same researcher worked with a team of two locally recruited and trained research assistants to interview families at their place of residence or community meeting points. Research assistant training included mock interviews to ensure appropriate data collection, followed by closely supervised interviews with research participants. After each interview, the lead researcher reviewed the data collection sheets to clarify any points of confusion with the research assistant and participant. Family interviews started in the rural area north of Momostenango and extended to the rural area south of Momostenango to ensure comprehensive data collection.

### 2.5. Ethics

The study was approved by the University of Saskatchewan Research Ethics Board. Since Indigenous people represent a vulnerable population, the research protocol specifically addressed confidentiality and employed a respectful approach. While obtaining participants’ consent, their rights and freedoms were fully explained. To assure participant confidentiality, data were only analyzed using north or south areas around Momostenango as locations instead of actual community names. All participant quotes have been anonymized.

### 2.6. Quantitative Analysis

Household food security scores were calculated using responses to five questions related to food security in the questionnaire.

1. In the past 6 months, have you worried about a lack of food in the household?; 

2. In the past 30 days, was there insufficient money in the household to buy food?; 

3. In the past 30 days, did a member of your household eat less than they wanted to because of lack of money?; 

4. In the last 30 days, did a member of your household skip breakfast, lunch, or dinner because of lack of money?;

5. In the last 30 days did a member of your household complain about hunger because of insufficient food in the household? 

Based on the scoring method outlined by Chaparro (2012), household food security scores were calculated by summing the positive responses to these questions. Scores could range from a low of 0 (all responses coded “no” or 0) to a high of 5 (all responses coded “yes” or 1) [20]. Food security categories were defined as follows: food secure (score of 0 or 1); moderately food insecure (score of 2 or 3, meaning unable to regularly eat healthy, nutritious diets); and severely food insecure (score of 4 or 5, meaning consuming an insufficient quantity of food) [21]. In addition to the above questions, mothers were also asked whether their households had to decrease consumption of certain foods in the last 6 months due to economic limitations. 

To characterize households in each food security category, descriptive statistics (i.e., means and frequencies) were calculated for selected variables and stratified by food security status. Chi-squared tests were used to assess differences between the food-secure group compared to the moderately food-insecure and severely food-insecure groups, with statistical significance reached if *p* < 0.05.

### 2.7. Qualitative Analysis

Detailed interview notes were translated into English by the interviewer. Inductive content analysis using the constant comparative approach guided analysis of the interview transcripts [22,23]. The analysis was conducted in three stages: (1) coding all the interview transcripts, using frequent comparison to establish preliminary themes, and building a coding grid, which was validated by 2 researchers; (2) reviewing the coding of all interviews with reference to the preliminary themes and identifying the most prominent themes related to food security; (3) finalizing themes and placing relevant data extracts under each theme, including areas of agreement and conflict within and across groups [24]. The final coded transcripts were reviewed and validated by the second researcher. 

Following the separate analysis of quantitative and qualitative data, the two data sources were examined for convergence and dissonance through use of a side-by-side chart with main data points organized under the pillars of food security. The integrated data were then reviewed to reveal new insights beyond those possible through a single method [25,26].

## 3. Results

### 3.1. Key Informant Interviews

Key informant interviews were held with agricultural group leaders in six communities, including five in rural areas and one in the city of Momostenango. Eight men and four women participated. Main themes that emerged from the data include the following: (1) subsistence agriculture, (2) commercial production, (3) challenges related to climate, capital, market, and capacity, and (4) sustainable opportunities.

### 3.2. Subsistence Agriculture

Respondents agreed that families with small plots of land usually engage in subsistence agriculture to “grow corn and beans for their families” (SB). Dependent on available resources, some families also have fruit trees, raise egg-laying hens, and cultivate cabbage, radishes, cucumber, and onion to feed their families.

### 3.3. Commercial Production 

Most respondents mentioned that families commonly generate income by cultivating vegetables and fruit to sell at the local market, including tomatoes, green beans, onions, peas, lemons, and oranges. A few families have developed the capacity to raise bees and harvest honey, which they sell to a coop. Others “raise chickens, pigs, rabbits and sheep to sell” (SB). One respondent mentioned gathering firewood and ocote (fire starter made by cutting bark and collecting sap) to sell and acknowledged that although this is profitable, it is not sustainable.

### 3.4. Challenges Related to Climate, Capital, Market, and Capacity

Respondents indicated that they live in a hot climate on the edge of the dry corridor, but more recently they have been experiencing increased drought and hail, resulting in crop failures. When it does rain, there is a lot of hail, resulting in severe crop losses. Some crops, such as “green beans, have ceased to grow due to climate change and drought” (PM).

Participants described not having sufficient fertilizer, water, and protective greenhouses to cultivate crops. Due to poor soil conditions, fertilizer is required to grow a bountiful crop. Many families have small plots of land but are limited to growing drought-resistant crops due to poor access to water. Although water is available underground, most families do not have pumps to extract the water, which would allow them to grow more diverse crops, including tomatoes, oranges, and avocados. Greenhouses to protect crops from pests and hail are cost-prohibitive for most families. There is also interest in hen and egg operations, but this requires capital to construct fencing to protect them from dogs and wild animals. Overall, obtaining capital to purchase agricultural inputs is a challenge.

Cheap Mexican imports, such as eggs and tomatoes, are creating a challenge because they are sold for a low price. Families find they cannot make a profit selling at these prices. There has also been some developmental work to start a youth network that grows mushrooms; however, there is “not sufficient market demand for mushrooms” (MM).

Some respondents recognized that poor education is the root of malnutrition. Some families grow small quantities of coffee but have experienced difficulties with disease and need training to support this venture. Although most respondents belong to loosely defined agricultural organizations, none are official cooperatives due to difficulties with navigating the registration process.

### 3.5. Sustainable Opportunities

Most respondents expressed interest in diversifying their income sources, and several have engaged in sustainable projects. One family has developed a small tourism business, Turicentro Kachelaj, with the aim of “generating sustainable employment, and protecting the forest and natural environment” (LC). However, they need capital to grow and progress over the long term.

Others are interested in raising turkeys and producing turkey eggs. Community members have the capacity to provide guidance on turkey care, common illnesses, and vaccinations. Turkeys can be sold in December to raise funds to purchase school supplies that are needed in January, and the eggs can be sold year-round. They already have a demonstration hen-and-egg operation to raise community capacity, and noted that when they have the resources, “80% of women are successful with home hen and egg operations” (LC). Some respondents suggested growing more fruit trees using natural fertilizer from chicken waste.

Participants indicated that they had attended trainings on nutrition and agriculture provided by the Ministry of Agriculture. They expressed interest in “trainings on craft production” (XQ), cultivation and commercialization of citrus fruits, and orientation to natural medicine. They have women’s groups and agricultural associations that can assist with organizing training sessions.

### 3.6. Family Interviews

**Figure 2:**
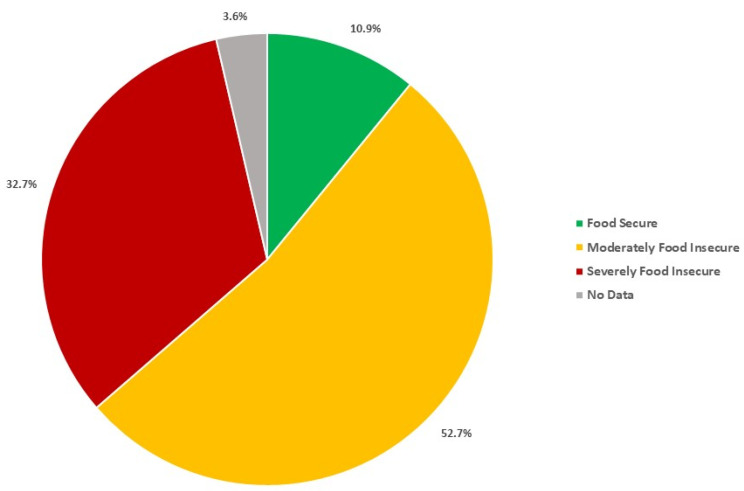
Food security status (*n* = 53).

Fifty-five mothers from 13 distinct communities around and within the city of Momostenango consented to be interviewed. Of the 53 households that responded to the food security questions, 85.4% were food insecure (Figure 2). As outlined in Table 1, the only significant demographic difference between food-secure, moderately food-secure, and severely food-insecure respondents was the reason for leaving or not attending school during youth, with food-insecure respondents commonly leaving school due to economic limitations, while food-secure respondents often left school due to marriage or pregnancy. Although not significantly different, a high percentage of severely food-insecure respondents reported having never attended school (41.2%). The most common main source of income for food-insecure families was agricultural labor, while for food-secure families it was other day labor and small business sales.

The majority of households (92.7%) were engaged in some form of agriculture (Table 2). Food-secure families only reported working their own or leased land, while food-insecure families most commonly worked their own land in addition to engaging in day labor. Corn was the most common crop, cultivated by 89.1% of households, followed by beans, at 80.0% of households, and other vegetables and fruits. Very few households cultivated coffee or mushrooms. Some food-insecure families engaged in fishing or collection of wild fruits, most commonly for family consumption, with the highest proportion of severely food-insecure families collecting wild fruits (33.3%) (Table 2).

About half of both food-secure and food-insecure households generated income from the sale of basic grains and chickens (Table 2). The sale of other crops, pigs, and eggs was also fairly popular among food-secure and moderately food-insecure households, however, less so among severely food-insecure households. 

All families reported experiencing one or more adverse events, such as family member deaths and job losses, over the past year that impacted their access to food (Table 3). Although not significant, a high proportion of moderately food-insecure families (69.0%) reported agricultural losses due to drought, pests, or poor animal health resulting in decreased access to food.

During periods of food scarcity, families reported employing several strategies, including altering food use, reducing expenses, and generating funds (Table 3). Selling small animals and asking family for help were common coping strategies among all groups. Borrowing funds to buy food was also fairly popular among severely food-insecure (22.2%) and moderately food-insecure families (13.8%). Removing children from school was a strategy employed by some moderately and severely food-insecure families (3.4% and 16.7%, respectively). In terms of food use, severely food-insecure families were significantly more likely to reduce portion sizes (72.2%) compared to moderately food-insecure (34.5%), while none of the food-secure households reported reducing portion sizes (Table 3). Food-secure families only reported eating less preferred foods. Eighty percent of households reported consuming less meat during periods of scarcity. Moderately food-insecure families were significantly more likely to report consuming less meat (93.1%) compared to severely food-insecure (83.3%) and food-secure (33.3%) households (Table 3). A reduced intake of beans was only reported by four severely food-insecure households (22.2%).

Six households declared that one or more family members had migrated to Guatemala City or the United States, primarily in search of employment (Table 3). Families reported receiving remittances from half of the migrants.

When presented with a list of potential food security interventions, a high proportion of respondents indicated interest in hen-and-egg operations (96.4%) and high-protein or climate-adapted corn (74.5%) to enhance their food security. A smaller proportion were interested in craft training (36.4%) or other options (Table 4).

There was a high degree of convergence between the qualitative key informant and quantitative family interview data strands related to findings under all four pillars of food security (Table 5).

Quantitative and qualitative data strands highly coincided in relation to concerns about food availability, food accessibility, and stability. However, there was silence from the key informants on several points related to food use noted from the quantitative family interview data. Food use in response to food scarcity was not probed during the key informant interviews. All data were integrated into a conceptual framework about the experience of food insecurity (the graphical abstract).

## 4. Discussion

This is the first study to evaluate food security using a comprehensive exploratory sequential mixed-methods study design in Momostenango, Guatemala. Overall, 85% of families interviewed were food-insecure. All respondents resided in rural areas that have traditionally struggled with food security. Overall, the results suggest that food-insecure households are characterized by being larger, having an older mother with less education due to economic restraints during childhood, and deriving most family income from agricultural day labor. This suggests that food insecurity in rural areas is a long-term structural phenomenon associated with limited family resources that reproduces itself in subsequent generations.

The national government has not prioritized investments in basic healthcare and social services, development initiatives, technology, cultural preservation, and gender equity, which has served to limit Mayans’ access to resources and further threaten their food security over the long term [27]. Government inaction to resolve these challenges jeopardizes the invaluable contribution that ancient agricultural knowledge could make to bolster food sovereignty and sustain local markets.

In addition, the uncontrolled export of food commodities serves to undermine dietary diversity by displacing the production and consumption of fresh, nutritious foods in rural areas [28]. Consequently, residents of rural Guatemalan agricultural communities share significant dietary concerns with the urban “food deserts” described in high-income countries. Addressing these structural issues regarding lack of government investment and regulations to support health food production is crucial to foster sustainable agricultural practices to overcome long-term food insecurity in rural Mayan communities.

During periods of food scarcity, food-secure families reported generating funds through the sale of small animals and asking family for help, and the dietary impact was usually limited to consuming less preferred foods and consuming less meat and dairy products. In comparison, the impact of periods of food scarcity was more profound on food-insecure families. Similar to food-secure families, moderately food-insecure households reported generating funds through the sale of small animals and asking families for help, in addition to borrowing funds for food, selling furnishings, and reducing expenses. As well as eating less preferred foods, moderately food-insecure families reported reducing portion sizes, eating fewer meals, and not eating for entire days. They were significantly more likely to consume less meat compared to other groups and commonly reduced intake of breads and cereals, dairy products, and fruits and vegetables.

Severely food-insecure families experienced even more serious repercussions during periods of food scarcity. In addition to the strategies employed by moderately food-insecure families, a higher proportion of severely food-insecure households relied on borrowing funds for food. In terms of food use, they were significantly more likely to reduce portion sizes compared to other groups and commonly consumed fewer meals per day and did not eat for whole days. Severely food-insecure families reported consuming less foods from all food groups and notably were significantly more likely to consume less beans during periods of scarcity. A reduction in the consumption of beans, a traditional staple along with corn tortillas, indicates a pronounced state of severe food insecurity.

In comparison to data gathered through the use of a similar questionnaire, the prevalence of food insecurity may be increasing in the municipality of Momostenango, or the current study simply reached the most vulnerable in rural areas. Data analyzed from the 2008–2009 ENSMI survey found that 22.8% of families with children under 5 years of age in the Department of Totonicapán were food-secure, while 52.2% were moderately food-insecure, and 25.0% were severely food-insecure [20]. Chaparro (2012) concluded that food-insecure families in the Western Highlands were commonly larger and lived in rural areas, while adult women in these households were more likely to have a primary education or less, which is similar to the current study results [20].

While the Guatemalan government does subsidize some crop production to support food security, these resources do not reach all areas and are generally of low value. For example, the Agricultural Stipend program, delivered by the Ministry of Agriculture, Livestock, and Food (MAGA), provides a one-time payment of one thousand quetzals (USD 120) to farmers that implement soil conservation practices that increase productivity [29]. This program has only reached 7357 low-income Guatemalans who are dedicated to tilling the land, a very low proportion of the 180,000 Guatemalan farmers. It is unknown if any farmers in Totonicapán have accessed this program as it was only available in selected departments originally, which did not include Totonicapán. In addition, the Guatemalan government offers agricultural insurance, but terms and conditions are always in flux to meet current government interests, making it an unreliable support over the long term. Recent beneficiaries of this program did not include farmers in Totonicapán [30].

Participants’ strong interest in hen-and-egg operations and high-protein/climate-adapted corn compared to other options (Table 5) indicate potential avenues for improving food security in rural Momostenango. Both these options fit within traditional rural agrarian activities and can be managed by mothers of the household. Jat et al. (2016) suggest that climate adaptation and mitigation strategies need to be location-specific due to diverse agroclimatic regions [31]. Crop diversification can mitigate some of the risk associated with climate change, supporting improved food security and income generation among resource-poor farmers [32]. Superior resilient varieties can play an important role in crop diversification to help farmers adapt to climate change [33]. Semilla Nueva is actively developing and promoting the use of biofortified corn in Guatemala [34]. They recognize the importance of improving upon traditional crops that have been cultivated for generations. One of the challenges with implementing the suggested food security interventions in Totonicapán is that the residents that could benefit most from these interventions do not have the economic capital to get started. The interventions would require some seed capital from a development agency or other organization to get started. The community has a history of working cooperatively to exchange their products among members, which would support long-term sustainability after the initial investment [35].

There is limited evidence that links egg consumption with improved child food security or nutrition outcomes. In Ethiopia, increased egg and eggshell powder intake by children under two years of age was significantly associated with improved health and nutrition outcomes, including reduced underweight and anemia [36]. In Ecuador, providing an egg a day resulted in significantly reduced stunting and underweight among infants and young children [37]. Accordingly, both adapted corn seed and household hen-and-egg operations may be viable routes to improving family food security and children’s nutritional health outcomes.

In terms of strengths and limitations, this study combines both qualitative and quantitative data to provide a comprehensive multidimensional analysis of food security among a highly marginalized Indigenous rural population. The study supports an overall characterization of food insecurity among this group through integrating lived experience perspectives with survey responses. This characterization of food insecurity is localized to rural areas of Totonicapán and is not necessarily generalizable to other areas of Guatemala. The cross-sectional nature of the survey limits any capacity to establish causality of food insecurity among this population, although the findings are in agreement with other limited evidence. Snowball sampling used to recruit participants may have introduced some bias, although participants were reached in the various rural areas surrounding Momostenango.

## 5. Conclusions

Food insecurity is a daily struggle for 85% of families in the rural areas of the municipality of Momostenango, department of Totonicapán. Present-day household food insecurity was associated with the mother of the family having abandoned school early in her youth due to economic restraints during childhood. This suggests that food insecurity is a self-replicating structural phenomenon among the rural Indigenous population, rooted in poor educational attainment linked to challenges with attending schools that are often a fair distance away, which ultimately serves as a barrier to a family’s further economic advancement. Both household hen-and-egg operations and adapted corn seed are viewed by the community as viable options to improve food security.

## Figures and Tables

**Table 1:** Participant demographics.

Respondent Characteristics	All (*n* = 55)	Food Secure (*n* = 6)	Moderately Food Insecure (*n* = 29)	Severely Food Insecure (*n* = 18)
**Age**				
Mean Age (years)	35.0	30.5	35.3	34.2
19–26 years	15 (27.3%)	2 (33.3%)	7 (24.1%)	5 (27.8%)
27–34 years	15 (27.3%)	2 (33.3%)	9 (31.0%)	4 (22.2%)
35–42 years	15 (27.3%)	2 (33.3%)	7 (24.1%)	6 (33.3%)
43–79 years	10 (18.2%)	0	6 (20.7%)	3 (16.7%)
**Family size**				
Mean size	6.7	5.0	7.1	6.7
2–4 members	13 (23.6%)	3 (50.0%)	7 (24.1%)	2 (11.1%)
5–6 members	14 (25.5%)	2 (33.3%)	7 (24.1%)	4 (22.2%)
7–8 members	16 (29.1%)	1 (16.7%)	6 (20.7%)	9 (50.0%)
9–13 members	12 (21.8%)	0	9 (31.0%)	3 (16.7%)
**Mother tongue**				
Kiche	52 (94.5%)	5 (83.3%)	27 (93.1%)	18 (100.0%)
Spanish	2 (3.6%)	1 (16.7%)	1 (3.4%)	0
**School achievement**				
Mean school (years)	2.4	3.2	2.4	2.1
No school	13 (23.6%)	1 (16.7%)	5 (17.9%)	7 (41.2%)
1–3 years	14 (25.5%)	0	10 (35.7%)	4 (23.5%)
4–6 years	15 (27.3%)	2 (33.3%)	9 (32.1%)	4 (23.5%)
7–12 years	9 (16.4%)	3 (50.0%)	4 (14.3%)	2 (11.8%)
Mother’s reason for leaving or not attending school *				
Economic limitations/could not pay registration	26 (47.3%)	1 (20.0%)	15 (55.6%)	10 (62.5%)
Married or pregnant	8 (14.5%)	4 (80.0%)	3 (11.1%)	1 (6.3%)
Not permitted to attend	4 (7.3%)	0	4 (14.8%)	0
Had to work or help family	3 (5.5%)	0	2 (7.4%)	1 (6.3%)
Bad grades or disliked	4 (7.3%)	0	1 (3.7%)	3 (18.8%)
Other reasons or do not know	3 (5.5%)	0	2 (7.4%)	1 (6.3%)
**Household Characteristics**				
**Community**				
Rural northwest	18 (32.7%)	3 (50.0%)	10 (34.5%)	5 (27.8%)
Rural southeast	32 (58.2%)	1 (16.7%)	16 (55.2%)	13 (72.2%)
Urban Momostenango	5 (9.1%)	2 (33.3%)	3 (10.3%)	0
**Main household income source**				
Agricultural day laborer	20 (36.4%)	0	10 (34.5%)	8 (44.4%)
Other day laborer	16 (29.1%)	2 (33.3%)	8 (27.6%)	6 (33.3%)
Small business sales	5 (9.1%)	2 (33.3%)	2 (6.9%)	1 (5.6%)
Arts and crafts	4 (7.3%)	1 (16.7%)	2 (6.9%)	1 (5.6%)
Salaried work	3 (5.5%)	1 (16.7%)	2 (6.9%)	0
Trades person	2 (3.6%)	1 (16.7%)	1 (5.6%)	0
Domestic laborer	2 (3.6%)	1 (3.4%)	2 (6.9%)	0
Small livestock/product sales	2 (3.6%)	0	1 (3.4%)	1 (5.6%)
**Main household income earner**				
Men	35 (63.6%)	4 (66.7%)	18 (62.1%)	11 (61.1%)
Women	11 (20%)	1 (16.7%)	7 (24.1%)	3 (16.7%)
Both	5 (9.1%)		3 (10.3%)	2 (11.1%)

* indicates significant difference between food secure, moderately food insecure and severely food insecure through x^2^ at *p* < 0.05.

**Table 2:** Agricultural engagement.

Agricultural Engagement	All (*n* = 55)	Food Secure (*n* = 6)	Moderately Food Insecure (*n* = 29)	Severely Food Insecure (*n* = 18)
Engaged in agriculture	51 (92.7%)	5 (83.3%)	27 (93.1%)	17 (94.4%)
**Type of engagement**				
Work own land and day laborer	22 (40.0%)	0	12 (41.4%)	9 (50.0%)
Work own land	21 (38.2%)	4 (66.7%)	11 (37.9%)	6 (33.3%)
Work leased land	6 (10.9%)	1 (16.7%)	3 (10.3%)	1 (5.6%)
Day laborer	2 (3.6%)	0	1 (3.4%)	1 (5.6%)
** Crops cultivated**				
**Corn**	49 (89.1%) ^	5 (83.3%)	26 (89.7%)	16 (94.1%)
Corn for family consumption	34 (61.8%)	3 (50.0%)	18 (62.1%)	12 (66.7%)
Corn for consumption and sale	5 (9.1%)	1 (16.7%)	3 (10.3%)	1 (5.6%)
**Beans**	44 (80%)	4 (66.7%)	25 (86.2%)	15 (83.3%)
Beans for family consumption	30 (54.5%)	2 (33.3%)	17 (58.6%)	11 (61.1%)
Beans for consumption and sale	5 (9.1%)	1 (16.7%)	3 (10.3%)	1 (5.6%)
**Vegetables and fruit**				
***Ayote (Squash)***	21 (38.2%)	2 (33.3%)	13 (44.8%)	6 (33.3%)
Family consumption	15 (27.3%)	0	10 (34.5%)	5 (27.8%)
Consumption and sale	4 (7.3%)	1 (16.7%)	2 (6.9%)	1 (5.6%)
***Chilacayote (Squash)***	19 (34.5%)	4 (66.7%)	10 (34.5%)	5 (27.8%)
Family consumption	14 (25.5%)	2 (33.3%)	7 (24.1%)	5 (27.8%)
Consumption and sale	2 (3.6%)	1 (16.7%)	1 (3.4%)	0
***Fruit trees/pineapples/bananas***	19 (34.5%)	2 (33.3%)	11 (37.9%)	6 (33.3%)
Family consumption	11 (20.0%)	1 (16.7%)	7 (24.1%)	4 (22.2%)
Consumption and sale	3 (5.5%)	1 (16.7%)	0	2 (11.1%)
***Chiles***	16 (29.1%)	3 (50.0%)	10 (34.5%)	3 (16.7%)
Family consumption	10 (18.2%)	1 (16.7%)	6 (20.7%)	3 (16.7%)
Consumption and sale	2 (3.6%)	1 (16.7%)	1 (3.4%)	0
***Other assorted vegetables***	27 (49.1%)	3 (50.0%)	17 (58.6%)	7 (38.9%)
Family consumption	18 (32.7%)	2 (33.3%)	11 (37.9%)	5 (27.8%)
Consumption and sale	3 (5.5%)	0	1 (3.4%)	2 (11.1%)
***Coffee (Family consumption)***	1 (1.8%)	0	1 (3.4%)	0
***Mushrooms (Family consumption)***	2 (3.6%)	0	1 (3.4%)	1 (5.6%)
**Wild foods collected**				
** Hunting**	0	0	0	0
**Fishing**	7 (12.7%)	0	5 (17.2%)	2 (11.1%)
Family consumption	5 (9.1%)	0	3 (10.3%)	2 (11.1%)
Consumption and sale	2 (3.6%)	0	2 (6.9%)	0
**Wild fruits**	10 (18.2%)	0	4 (13.8%)	6 (33.3%)
Family consumption	8 (14.5%)	0	2 (6.9%)	6 (33.3%)
Consumption and sale	1 (1.8%)	0	1 (3.4%)	0
**Agricultural products sold**				
Grains (corn, beans)	27 (49.1%)	3 (50.0%)	16 (55.2%)	8 (44.4%)
Other crops	10 (18.2%)	2 (33.3%)	7 (24.1%)	1 (5.6%)
Cows/goats	3 (5.5%)	0	2 (6.9%)	1 (5.6%)
Pigs	13 (23.6%)	2 (33.3%)	7 (24.1%)	4 (22.2%)
Chickens/turkeys	27 (49.1%)	3 (50.0%)	15 (51.7%)	9 (50.0%)
Eggs	15 (27.3%)	2 (33.3%)	11 (37.9%)	2 (11.1%)
Fish	1 (1.8%)	0	1 (3.4%)	0
** Other products sold**				
Arts/crafts	5 (9.1%)	2 (33.3%)	2 (6.9%)	1 (5.6%)
Prepared food	2 (3.6%)	0	1 (3.4%)	1 (5.6%)

^ 100% of those with land to cultivate.

**Table 3:** Problems encountered and food security coping strategies.

Problems Encountered over the Last Year	All (*n* = 55)	Food Secure (*n* = 6)	Moderately Food Insecure (*n* = 29)	Severely Food Insecure (*n* = 18)
Loss or lack of work ^	24 (43.6%)	3 (50.0%)	13 (44.8%)	7 (38.9%)
Sickness or death in the family ^	23 (41.8%)	3 (50.0%)	9 (31.0%)	10 (55.6%)
Rising food/transport prices ^	24 (43.6%)	3 (50.0%)	15 (51.7%)	6 (33.3%)
Agricultural losses due to drought, pests, and animal health ^	34 (61.8%)	3 (50.0%)	20 (69.0%)	10 (55.6%)
Debt or loss of home ^	7 (12.7%)	0	5 (17.2%)	2 (11.1%)
**Food Security Coping Strategies**	**All (*n* = 55)**	**Food Secure (*n *= 6)**	**Moderately Food Insecure (*n* = 29)**	**Severely Food Insecure (*n* = 18)**
**Alter food use**				
Eat less preferred foods	35 (63.6%)	3 (50.0%)	21 (72.4%)	10 (55.6%)
Reduce food portions *	23 (41.8%)	0 *	10 (34.5%) *	13 (72.2%) *
Eat fewer meals per day	11 (20.0%)	0	4 (13.8%)	6 (33.3%)
Do not eat the whole day	4 (7.3%)	0	3 (10.3%)	1 (5.6%)
**Reduce expenses**				
Reduce agricultural expenses	8 (14.5%)	0	5 (17.2%)	3 (16.7%)
Reduce health costs	7 (12.7%)	1 (16.7%)	3 (10.3%)	3 (16.7%)
Remove children from school	4 (7.3%)	0	1 (3.4%)	3 (16.7%)
**Generate funds**				
Sell small animals	17 (30.9%)	2 (33.3%)	9 (31.0%)	6 (33.3%)
Ask family for help	12 (21.8%)	2 (33.3%)	6 (20.7%)	4 (22.2%)
Borrow or use credit for food	8 (14.5%)	0	4 (13.8%)	4 (22.2%)
Sell furnishings	6 (10.9%)	1 (16.7%)	4 (13.8%)	1 (5.6%)
Migrate to look for work	6 (10.9%)	0	4 (13.8%)	2 (11.1%)
Ask institutions for help	5 (9.1%)	1 (16.7%)	2 (6.9%)	2 (11.1%)
Start small business	3 (5.5%)	0	1 (3.4%)	2 (11.1%)
Look for work	1 (1.8%)	0	1 (3.4%)	0
**Foods eaten less**				
Meat/poultry/fish/eggs *	44 (80.0%)	2 (33.3%) *	27 (93.1%) *	15 (83.3%) *
Bread and cereals	20 (36.4%)	1 (16.7%)	10 (34.5%)	9 (50.0%)
Dairy products	18 (32.7%)	2 (33.3%)	11 (37.9%)	5 (27.8%)
Vegetables/fruit	15 (27.3%)	0	11 (37.9%)	4 (22.2%)
Beans *	4 (7.3%%)	0 *	0 *	4 (22.2%) *
**Migration**				
Households with at least one family member who migrated	6 (10.9%)	1 (16.7%)	3 (10.3%)	2 (11.1%)
Second family member migrated from the same household	3 (5.5%)	1 (16.7%)	1 (3.4%)	1 (5.6%)
Third family member migrated from the same household	1 (1.8%)	0	1 (3.4%)	0
**Migrants**	***n* = 10**	***n* = 2**	***n* = 5**	***n* = 3**
**Migration reasons**				
Find work	9 (90%)	2 (100%)	4 (80.0%)	3 (100%)
Studying	1 (10%)	0	1 (20.0%)	0
Send remittance to family	5 (50%)	0	3 (60.0%)	2 (66.7%)

^ 100% of households that experienced these problems said that it impacted access to food. * indicates significant difference between food secure, moderately food insecure and severely food insecure through x^2^ at *p* < 0.05.

**Table 4:** Interest in interventions to improve food security.

Interest in Interventions to Improve Food Security	All (*n* = 55)	Food Secure (*n* = 6)	Moderately Food Insecure (*n* = 29)	Severely Food Insecure (*n* = 18)
Hen-and-egg operation	53 (96.4%)	5 (83.3%)	28 (96.6%)	18 (100.0%)
Corn (high-protein or adapted to climate)	41 (74.5%)	3 (50.0%)	23 (79.3%)	15 (83.3%)
Craft training	20 (36.4%)	3 (50.0%)	9 (31.0%)	8 (44.4%)
Mushroom cultivation	13 (23.6%)	0	8 (27.6%)	5 (27.8%)
Health training	12 (21.8%)	1 (16.7%)	5 (17.2%)	6 (33.3%)
Micronutrients	10 (18.2%)	1 (16.7%)	3 (10.3%)	4 (22.2%)
Loans	8 (14.5%)	1 (16.7%)	4 (13.8%)	3 (16.7%)
Solar panels	7 (12.7%)	1 (16.7%)	5 (17.2%)	1 (5.6%)
Water access	7 (12.7%)	1 (16.7%)	3 (10.3%)	3 (16.7%)
Technical specialist access	7 (12.7%)	1 (16.7%)	3 (10.3%)	3 (16.7%)
Business training	6 (10.9%)	1 (16.7%)	2 (6.9%)	3 (16.7%)
Family planning training	6 (10.9%)	1 (16.7%)	3 (10.3%)	2 (11.1%)

**Table 5:** Convergence between data strands.

Key Qualitative Observations	Key Quantitative Observations	Convergence between Strands		
		Agreement	Silence by One Strand	Dissonance
Food Availability				
Limited resources to produce commercial crops or raise livestock	High prevalence of food insecurity (85.4%)	X		
Malnutrition due to poor education	Among the severely food-insecure group, 63% left school early during youth due to economic limitations.	X		
**Food Access**				
Families generate income through selling agricultural products at local markets.	About half of all families generate income by selling grains, chickens, or turkeys at local markets.	X		
	About a third of all families generated funds for food by selling small animals.			
Cheap Mexican imports reduce demand for foods produced in Guatemala.	Only 11% of severely food-insecure families generated income from egg sales compared to food-secure families (33%) and moderately food-insecure families (38%).	X		
Opportunities to generate income include tourism, hen-and-egg operations, crafts, citrus fruit production, and natural medicine.	Strong interest from all families to enhance food security through hen-and-egg operations (96%), cultivating new varieties of corn (75%), and producing crafts (36%)	X		
	11% of households had at least one family member that migrated, usually to look for work. In total, 50% of individuals that had migrated sent remittances to their families.		X	
Malnutrition due to poor education	A total of 17% of severely food-insecure families removed children from school due to economic difficulties compared to moderately food-insecure (3%) and food-secure (0%) families.	X		
**Food Use**				
Families with land commonly grow corn and beans to feed themselves.	In total, 71% of families grew corn to feed their families; 64% grew beans to feed their families.	X		
	Consuming less animal products to cope with food insecurity was more common among the moderately food insecure (93%) and severely food insecure (83%) compared to the food secure (33%).		X	
	To cope with food insecurity, 22% of severely food-insecure families reported consuming less beans, while none of the other families did.		X	
	To cope with food insecurity, severely food-insecure families more commonly reported reducing portion sizes (72%) compared to moderately food-insecure (35%) and food-secure families (0%).		X	
**Stability**				
Crop failures due to drought and hail	High prevalence of food insecurity (85.4%)	X		
	Moderately food-insecure families reported the highest rate of agricultural losses (69%).			

## Data Availability

The data presented in this study are available on request from the corresponding author. The data are not publicly available due to privacy and ethical considerations.
